# Consequences of arthropod community structure for an at-risk insectivorous bird

**DOI:** 10.1371/journal.pone.0281081

**Published:** 2023-02-10

**Authors:** Cee S. Nell, Riley Pratt, Jutta Burger, Kristine L. Preston, Kathleen K. Treseder, Dana Kamada, Karly Moore, Kailen A. Mooney

**Affiliations:** 1 Department of Ecology & Evolutionary Biology and Center for Environmental Biology, University of California, Irvine, CA, United States of America; 2 California State Parks, San Clemente, CA, United States of America; 3 Irvine Ranch Conservancy, Irvine, CA, United States of America; 4 Natural Communities Coalition, Irvine, CA, United States of America; Sichuan University, CHINA

## Abstract

Global declines in bird and arthropod abundance highlights the importance of understanding the role of food limitation and arthropod community composition for the performance of insectivorous birds. In this study, we link data on nestling diet, arthropod availability and nesting performance for the Coastal Cactus Wren (*Campylorhynchus brunneicapillus sandiegensis*), an at-risk insectivorous bird native to coastal southern California and Baja Mexico. We used DNA metabarcoding to characterize nestling diets and monitored 8 bird territories over two years to assess the relationship between arthropod and vegetation community composition and bird reproductive success. We document a discordance between consumed prey and arthropod biomass within nesting territories, in which Diptera and Lepidoptera were the most frequently consumed prey taxa but were relatively rare in the environment. In contrast other Orders (e.g., Hemiptera, Hymenoptera)were abundant in the environment but were absent from nestling diets. Accordingly, variation in bird reproductive success among territories was positively related to the relative abundance of Lepidoptera (but not Diptera), which were most abundant on 2 shrub species (*Eriogonum fasciculatum*, *Sambucus nigra)* of the 9 habitat elements characterized (8 dominant plant species and bare ground). Bird reproductive success was in turn negatively related to two invasive arthropods whose abundance was not associated with preferred bird prey, but instead possibly acted through harassment (*Linepithema humile; Argentine ants*) and parasite transmission or low nutritional quality (*Armadillidium vulgare*; "pill-bug"). These results demonstrate how multiple aspects of arthropod community structure can influence bird performance through complementary mechanisms, and the importance of managing for arthropods in bird conservation efforts.

## Introduction

Birds are a charismatic and ecologically important component of biodiversity, and reports of their global decline are thus especially alarming [1]. Global change may act directly upon birds in numerous ways, including habitat loss [2], the physiological effects of warming and drought [3], increased disturbance [[4, 5]; extreme events], behavioral disruption [[6–8]; noise, light, glass] and hunting [[9, 10]; food, pet trade]. Importantly, global change may have cascading and indirect effects of equal or greater importance to such direct effects. These indirect effects include global change impacts on food resources [11], competition with invasive species [12], disease ([13]; Hawaii malaria) and predation [14]. Birds provide key ecosystem services and economic value as predators [15, 16], seed dispersers, insect control [17] and pollinators [18]. Birds also represent a source of inspiration in most human cultures [19]. Efforts aimed at bird conservation thus carry significant socio-economic benefits. The critical endeavor of bird conservation will require a mechanistic understanding of the factors driving their decline.

Most bird species rely on arthropods for some or all of their diet, and evidence for food limitation in birds suggests that observed decline in arthropod abundance [20] may contribute to bird declines. Food limitation is broadly recognized to be a key determinant of avian productivity [21, 22] and proposed to drive fundamental trade-offs in life-history evolution and thus bird diversity and trait variation [23, 24]. Research on the role of food limitation for birds has traditionally focused on Lack’s Winter Food Limitation Hypothesis [25], which proposes that higher clutch size in temperate (vs. tropical) habitats is due to greater availability of arthropod prey abundance in winter. Evidence supporting this hypothesis is somewhat limited [26] but includes the observations that temperate birds have greater prey capture rates (e.g. [27]) and increased nest attentiveness (e.g. [28]), and winter food supplementation can increase fecundity (e.g. [29]). More broadly, food availability and distribution have been observed to drive bird foraging [30, 31] and provide important environmental cues that can influence bird reproductive efforts in terms of nesting location, mate choice, investment in offspring, and ultimately population size [16, 21]. Recent studies documenting dramatic declines in arthropod abundance [32, 33] suggest that food availability may underline parallel global declines of insectivorous birds [34].

The drivers of change in arthropod communities that may affect insectivorous birds are poorly understood, but several underlying mechanisms have received consideration. Increased aridity [35, 36] and insecticide use [32] have each been linked to arthropod decline. Changes in arthropod community composition may be of equal importance as arthropod abundance does not necessarily correspond to the amount of food available for a foraging bird [37]. Birds prefer certain prey groups based upon prey quality [38, 39], encounter rate and handling time [40]. Factors affecting arthropod community composition include the presence of invasive plants [41, 42] and arthropods [43], drought [35, 36], nitrogen deposition [44] and habitat fragmentation [45]. In addition, invasive arthropods may directly harm birds through harassment and predation [46, 47] or parasite transmission [48, 49].

Assessing global change effects on arthropods and insectivorous birds thus requires understanding the importance (or role) of food limitation, the arthropod taxa driving bird performance, and the environmental factors affecting arthropod density and community composition. In this study, we sought to address these knowledge requirements for a bird population in decline. The Coastal Cactus Wren, *Campylorhynchus brunneicapillus sandiegensis*, is a year-round resident of the coastal sage scrub plant communities along the coast of southern California, U.S.A. and northern Baja California, Mexico [50]. Historically widespread, Coastal Cactus Wrens have been reduced to less than 20% of their original distribution and abundance [51, 52], including substantial declines within the boundaries of this region’s largest, relatively undisturbed reserves. In this study we used the following complementary approaches: (i) we characterized arthropod prey in Coastal Cactus Wren nestling diet using DNA metabarcoding of fecal samples; (ii) we estimate arthropod biomass and community composition associated with the most common habitat elements in occupied Coastal Cactus Wren territories; and finally (iii) we assessed the relationships between bird productivity and arthropod community composition. By considering Coastal Cactus Wren conservation in terms of their dietary preferences and foraging environment, we highlight opportunities for improved management of this and other species through the lens of multi-trophic interactions.

## Methods

### Study system

The Coastal Cactus Wren is a year-round resident of the coastal sage scrub plant communities (CSS) along the coast of southern California and northern Baja California [51, 53]. Coastal Cactus Wrens were historically widespread and abundant in this region but have declined over the past several decades [53]. It is designated as Species of Special Concern by the California Department of Fish and Wildlife, and coastal populations are target species for regional conservation programs [51].

During the breeding season Coastal Cactus Wrens nest in *Opuntia littoralis* and *Cylindropuntia prolifera* cacti [50] and occupy well-defined territories that encompass their primary foraging habitat. They forage for insects both on the ground and from plant canopies [54] and their diet is composed of invertebrates (~70%), occasional fruit and seeds, and small lizards [55]. The breeding season is characterized by a relatively long nesting cycle: nest building to fledgling takes 42–55 days, after which young birds remain in the nesting territory and dependent on their parents for an additional 17–25 days [53]. Breeding takes place from late February or early March through June, with first nestlings hatching as early as the first week of March [54]. Mated pairs build multiple nests throughout the breeding season, typically within the same territory (0.4 to 1.4 ha), and commonly use them to incubate different clutches. On average, mating pairs produce 4 eggs per clutch that take 16 days to hatch, however clutch size, clutch survival, and other reproductive behaviors can be influenced by food availability [56].

### Focal territories and reproductive monitoring

**Fig 1:**
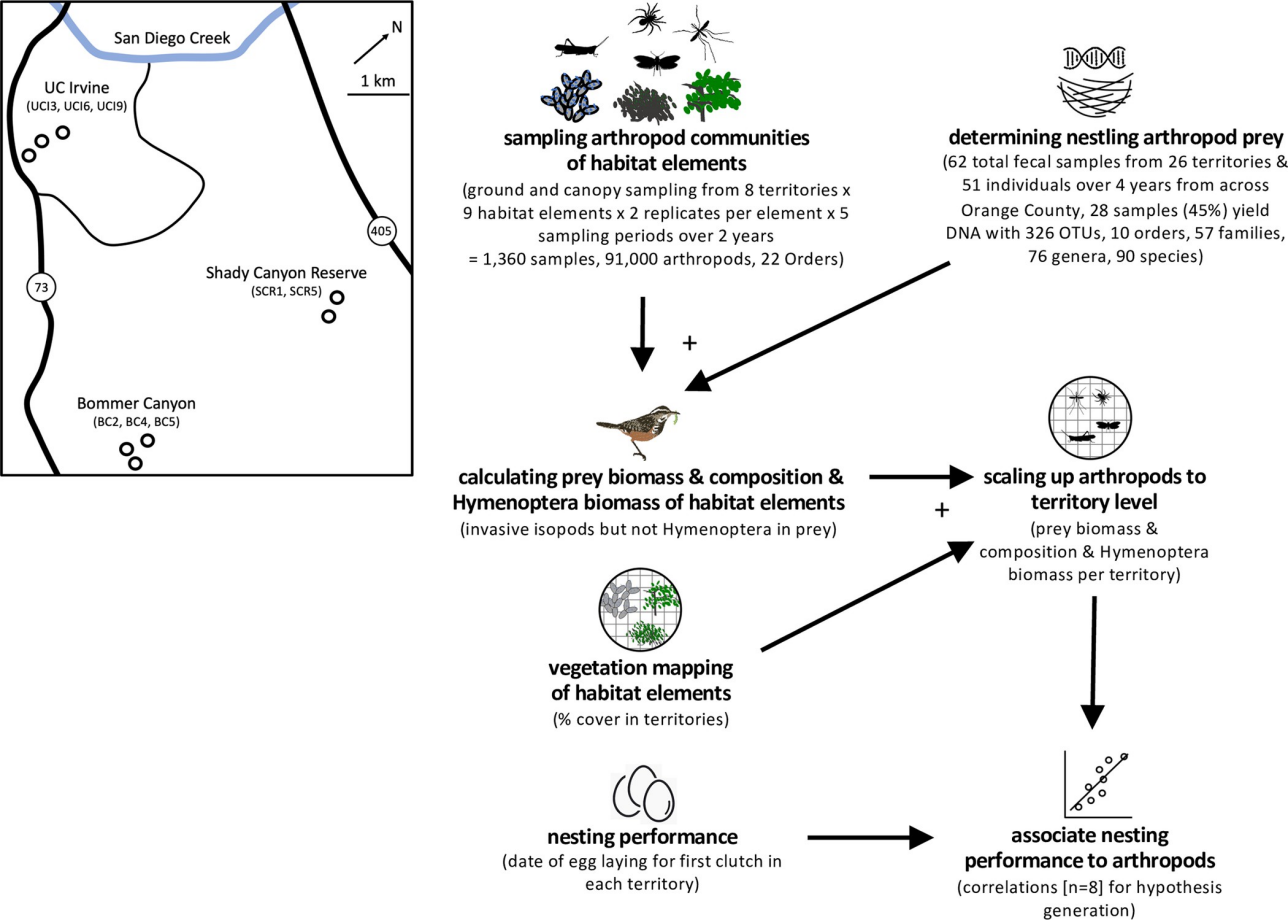
Overview of experimental design. The map identifies the location of the eight studied Cactus Wren territories within Orange County. The flow chart indicates the different data sources and sample sizes and how they are integrated in analyses. Details on each data stream and analyses procedures are provided in Methods text.

In 2012 we selected eight occupied territories distributed among three sites, 4–5 km apart in Orange County, California, with 2 or 3 territories within each site separated by 200–600 m (Fig 1). Bird productivity was tracked in 2012, where nests were visited at least once a week during the breeding season (March through July) to color band birds and monitor nesting attempts, dates of first egg, the number of eggs and nestlings, and subsequent fledgling success. The date of the first clutch is strongly linked to the number of broods produced and increases fecundity in Coastal Cactus Wren (S1 Fig in S1 Appendix; [51]) and other song birds [57, 58]. Studies of other species have documented earlier egg lay dates and breeding success (number of fledglings) in response to food supplementation[59].

### Nestling arthropod diet analysis

A total of 62 fecal samples were collected from birds across Orange County to characterize arthropod prey in Coastal Cactus Wren diet using DNA metabarcoding, with 61 being nestlings and 1 being a fledgling ("nestlings" hereafter). These samples were collected opportunistically during nest monitoring efforts between 2010 and 2011 (n = 20) and 2012 and 2013 (n = 42) in which fresh fecal material was obtained directly from nestlings at banding, preserved in 80% ethanol in the field, and later stored in a -20° C freezer. We estimate that diet samples were sourced from at least 51 individuals in 26 different Coastal Cactus Wren territories in Orange County, CA, including 6 of the 8 territories that are the emphasis of this study. All nestlings were handled for banding and fecal sample collection in accordance with standard protocols [60]. These activities were authorized under Dana Kamada’s U.S. Master Banding Permit #22956, Scientific Collecting Permit (SC-001360), and Memorandum of Understanding with the California Department of Fish and Wildlife.

We used taxon-specific DNA metabarcoding methodology to identify arthropod prey from fecal samples collected from nestling and fledgling birds. Group‐specific PCR primer sets specify the range of taxa that produce an amplicon in a PCR [61, 62], which can be generalized to capture broad classes of organisms like arthropods [63]. This methodology has been successful in characterizing prey diversity from gut and fecal samples because amplification is possible with degraded samples or very small quantities of target DNA [63, 64].

In 2012, we used the FastDNA SPIN Kit for Feces (MP Biomedicals, Santa Ana, CA) to isolate DNA from samples following the manufacturer provided methodology. The concentration of DNA was measured to assess extraction success using a 50 bp DNA ladder (New England BioLabs), and diluted if necessary to 50 ng/μl. DNA extracts were then amplified using barcoded (MID labeled) forward (ZBJ-ArtF1c) and reverse (ZBJ-ArtR2c) primers (Eurofins) targeting 157 bp fragments of the mitochondrial cytochrome *c* oxidase 1 gene region (CO1) [63], designed to detect multiple classes of arthropod taxa. Approximately 100 ng of DNA extract (2 μl) from each fecal sample was added to a PCR cocktail (25 μl reaction) containing 12.5 μl of PCR ImmoMix (MyTaq HS Mix from Bioline, includes premixed Taq, PCR buffer, MgCl_2_, and dNTPs), 0.5 μl of both forward and reverse primers (final concentration in reaction 0.2 μM), 1.0 μl BSA (0.4 μM/μl; New England Biolabs, BSA-007), and 9.5 μl H_2_O. PCRs were carried out according to the following conditions: 95°C for 3 minutes followed by 49 cycles of 95°C for 30 seconds, 55°C for 25 seconds, 72°C for 15 seconds, followed by a final extension at 72°C for 10 minutes. For every 4–5 reactions a PCR blank was included as a negative control. 4 μl of each of the PCR products were then visualized using gel electrophoresis against a 50 bp ladder (New England BioLabs) to identify positive products of the expected size (157 bp). DNA was successfully amplified in 35 of 62 samples and purified using The Wizard SV Gel and PCR Clean-Up System (Promega). DNA concentration of each PCR product was then quantified on a NanoDrop Microvolume Spectrophotometer (ThermoFisher). PCR products were. diluted to equimolar concentration and pooled together. The pooled sample, containing 35 barcoded PCR products, was sequenced (by Beckman Coulter Genomics) using the 454 GS FLX platform (LibA/A) with Titanium chemistry (Roche-NGS) to generate an amplicon library containing 553K reads with MID tags and the A adaptor.

Qiime bioinformatics software [65] was used to assign raw multiplexed sequences to wren fecal samples and select arthropod Operational Taxonomic Units (OTUs). OTUs were defined at 97% similarity clustering threshold and compared to reference species-level barcode records in the BOLD database (accessed July 10, 2019) to obtain the closest match in identification [66]. Established cutoffs for percent similarity to BOLD sequences were used to designate the taxonomic resolution for each OTU, with matches above a threshold of 99.3% similarity designated at the species-level, 94.9% for genera, 91% for family, and 85.9% at the order [63]. We use regional knowledge of arthropod fauna to refine the assigned taxonomy where possible. For example, all OTUs identified as Armadillidiidae (Isopoda) were assigned at the species-level to *Armadillidium vulgare* because this is the only species occurring within the study location. When sequences matched to multiple unrelated taxa with equal similarity, classifications were resolved to the higher taxonomic rank that agreed among competing matches. Any sequences that were not matched at the order-level in the BOLD database, either due to low similarity or conflicting results, were compared to the top results returned by the NCBI GenBank database [67] and coarse patterns of phylogenetic clustering. Any remaining identifications unresolved at the order-level were excluded from subsequent analyses due to uncertainty. The OTU sequences and table produced are available upon request.

### Arthropod sampling

To characterize prey resources, we sampled arthropods in all eight focal territories (Fig 1). The arthropods in each territory were characterized from 9 habitat elements: bare ground, native grasses, non-native grasses, *Opuntia littoralis*, *Brassica nigra* (non-native), *Artemisia californica*, *Eriogonum fasciculatum*, *Rhus integrifolia*, and *Sambucus nigra*. Collectively these 9 habitat elements constitute most of each territory (74–98% of ground cover; see Vegetation Mapping below), with the remaining coverage consisting of other, relatively rare plants. Within each territory, two separate spatial blocks were selected that each contained a representative of each habitat element.

We sampled arthropods at three timepoints in 2012 (March 7- May 10, May 13 –June 4, July 2 –July 22) and twice in 2013 (February 12-February 21, April 22 –May 1), approximately corresponding to nest building (March 2012 and February 2013), incubation (May/June 2012 and April/May 2013) and fledging (July 2012 only). Canopy sampling was conducted via vacuum sampling for 3 minutes with a Bioquip backpack aspirator powered by a 5.1-amp suction motor pulling air at 4250 L/min through a 12 cm diameter nozzle fitted with exchangeable fine mesh bags. Bags were immediately placed into coolers and then transferred to -20° C freezers for storage. Vacuum sampling of plant canopies was complemented by visual searches for 3 minutes. Understory sampling was conducted via pitfall trapping with 7 cm diameter x 7 cm deep plastic cups buried flush with the soil surface, filled to a depth of 3 cm with soapy water and left in place for 48 hours. Bare ground was sampled by both pitfall sampling and vacuum collecting any arthropod observed within 0.5 m of the pitfall trap, with these two samples being referred as “ground” and “canopy”, respectively, for consistency with sampling terminology for the plant-based habitat elements. A few of the experimental blocks did not contain all the habitat elements in our design (native grass, *Rhus integrifolia*, *Sambucus nigra* each missing from 1–2 blocks; *Brassica nigra absent from subset of time points*).

The vast majority (99.84%) of arthropods from each sample were identified at least to the level of Order, counted, and measured for body length under a dissecting microscope. Within Hymenoptera we identified and separately counted *Linepithema humile* (Argentine ant) for a subset of samples, determining that they represented 95% of all individuals. Within Hemiptera we identified and separately counted the three sub-orders Heteroptera, Auchenorrhyncha, Sternorrhyncha. For visual searches of plant canopies, arthropods were identified, counted, measured in the field, and combined with vacuum sample data for analysis. Arthropod data were subsequently converted to arthropod biomass based upon published length-biomass relationships at the Order or suborder level [68]. Biomass estimates did not include arthropods identified as Raphidoptera, Archaeognatha, Pseudoscorpionida, Ephemeroptera, and Siphonoptera due to lack of size-biomass parameters, however it is improbable this affects our conclusions as they were rare in both the environment (0.5% of all arthropods) and diet (Siphonaptera detected in 7.14% of samples).

### Vegetation mapping

Each focal territory was defined by a 100 m circular buffer around an occupied nest and divided into smaller polygons for vegetation mapping (Fig 1). Within each polygon we estimated the percent cover of our focal habitat to the nearest 1%. Any non-target habitat elements that were greater than or equal to 10% of the total area in the polygon (or ~1% overall) were also recorded. Areas classified as bare ground included soil crust communities but not those covered in leaf litter. Because vegetation surveys were conducted after peak mustard (non-native Brassicaceae; BRSP) phenology, percent cover was estimated from dead inflorescences. Using the total measured area of each polygon, we calculate the percent cover of each habitat element at the territory-level.

### Data analysis

Our analyses sought to integrate data on (i) nestling diet, (ii) arthropod communities associated with each habitat element, (iii) the composition of each territory with respect to those habitat elements, and (iv) bird performance in each of those territories (Fig 1). These data sets allow for a robust comparison of nestling diet with arthropod communities associated with each habitat element, thus providing insight into which habitat elements are likely of greatest importance to reproductive success. We in turn use data to estimate territory-level variation arthropod communities and relate this to territory-level variation in bird performance. These analyses are by necessity limited to relatively small sample size (n = 8), and these findings must thus be interpreted with caution and taken as hypotheses requiring further investigation.

To describe the Coastal Cactus Wren nestling diet, we rank arthropod prey orders by the frequency (%) they were detected among the fecal samples with detectable arthropod DNA (45.1% of all samples, see below). We also considered the relative read abundance (RRA), number of OTUs, and species richness within each prey order as metrics for analysis. In exploratory analyses we found the outcomes to be qualitatively similar to their frequency in samples (S2 Table, S1 Fig in S1 Appendix). In addition, using the frequency of samples limits biases that can be attributed to differences in database coverage and reads among different taxa groups. We consider any arthropod order identified from more than 1 fecal sample to represent prey taxa in subsequent analyses (S2 Fig in S1 Appendix). In doing so, we exclude three arthropod orders occurring once in the diet: Hymenoptera, Psocoptera, and Siphonaptera. This decision is supported by the rarity of Psocoptera and Siphonaptera in arthropod samples (Siphonaptera likely reflect the consumption of avian parasites). In contrast, Hymenoptera (95% Argentine ants) were among the most abundant arthropods sampled from both canopy and ground samples and therefore an important dimension of the arthropod community at these sites. However, in the fledgling diet a single Hymenopteran OTU was identified as a Braconid wasp (S2 Table in S1 Appendix).

We tested for variation among habitat elements in biomass for each arthropod order and then extracted marginal means from these models for each habitat element, specific to each territory. Because of the predominance of Argentine ants in the environment and potential negative implications for native species, we choose to analyze the biomass of environmental Hymenoptera (95% Argentine ants; see above) as an independent axis and ask whether the biomass of invasive ants is associated with reduced prey resources within the nesting territory. We tested for variation in prey biomass, Hymenoptera biomass, and prey composition among habitat elements and territories. In all tests we analyze ground and canopy arthropod biomass separately due to inherent differences in the sampling methodology. First, we ran separate linear mixed models (LMMs) predicting prey biomass and Hymenoptera biomass using the ‘lme4’ package for R [69]. In these models we include habitat element, site, and territory (nested in site) as fixed effects with a full interaction structure among fixed effects. Time block and experimental block (nested in territory) were included as random effects in the initial models, but subsequently dropped if the variance associated with the random term was 0 to improve model convergence. All LMMs were executed with each habitat element sample as the replicate. A constant of 1 was added to biomass estimates and log-transformed to improve normality of the residuals. From these models we obtained territory-specific estimated marginal means of arthropod biomass for each order and habitat element using the ‘emmeans’ package for R [70]. For all tests, we employ a Poisson distribution to achieve normality of the residuals using count data for the response variables. We log-transformed the total biomass of non-native arthropods due to extreme values in some territories. Further, we examined variation in arthropod composition attributed to the main effects of habitat elements, site, and their interaction on arthropod composition using PERMANOVA tests for canopy and ground separately. Each test was based on pairwise Bray-Curtis dissimilarities between samples based on the estimated biomass of the prey orders in each territory. Order-level estimates of arthropod biomass were calculated as the mean per territory x habitat element x arthropod order by averaging across sampling blocks and totaling across all time blocks.

Next, we estimated arthropod biomass at the territory-level using the percent cover of each habitat element and order-level estimates of arthropod biomass for each territory (described above). For each habitat element, estimates of arthropod biomass were multiplied by habitat element percent cover in each territory. This was done separately for each prey order and based on territory-specific estimates of arthropod biomass to obtain territory-level estimates of composition. In this calculation we use the percent cover of habitat elements in each territory to determine the relative biomass of each arthropod order for an equal area. We then multiply relative arthropod biomass by a constant area, 31,416 m^2^ (the area of vegetation surveys), to approximate arthropod density at the territory-level. This conversion from vegetation cover to prey biomass is crude in that we imply that the equivalent of 1 m^2^ of cover for a given habitat element takes the same amount of time to sample arthropods (3 min) across all habitat elements. This is more or less true for several of our shrub species that are more similar in size and structure, however it is likely that relatively more area (ground cover) was sampled within this time frame and from habitat elements with less complex morphologies (e.g., grass and cacti).

Using these estimates, we characterize canopy and ground arthropods for each territory in terms of total prey biomass, Hymenoptera biomass, and prey composition. We quantify total arthropod prey biomass as the sum of prey orders (Araneae, Diptera, Coleoptera, Isopoda, Lepidoptera, and Orthoptera). As described above, Hymenoptera biomass was largely represented (95% of individuals) by a single invasive species, *Linepithema humile*, which is extremely dominant when present in this system. To quantify prey composition, we ran a PCA on the relative biomass of prey orders to reduce dimensionality, and use the first two PC axes to capture the breadth of variation in composition. At the territory-level, we use correlations (n = 8) to evaluate the relationships between arthropods and bird reproductive success using the date of the first egg produced (first egg date). We relate each first egg date and the number of fledglings to arthropod communities (prey biomass, Hymenoptera biomass, prey composition) with each territory as the unit of replication in generalized linear models.

## Results

### Bird monitoring

During the nesting season of 2012, mated pairs started to produce their first eggs between late February (Julian day 52) and early May (Julian date 130) (Table 1). Across territories, nesting pairs had a mean of 1.9 nest efforts (±0.35 SE), clutches of 3.3 eggs (±0.25 SE) per nest effort, and a seasonal total of 6.4 eggs (±1.39 SE). There was considerable mortality during chick development (Table 1) resulting in an average of 2.8 fledglings (±0.92 SE) per pair for the breeding season. The first egg date was correlated with the number of nesting attempts, eggs, and fledglings in each territory (S1 Fig in S1 Appendix).

**Table 1:** Outcome of Coastal Cactus Wren nesting efforts across territories monitored in 2012. Productivity was tracked among territories in terms of the date that the first egg was laid, the total number of nesting attempts, eggs laid, fledgling success, and the timing and causes of nesting failure.

Site	Territory	First egg date (julian)	Nest attempts	Successful Clutches	Total eggs	Fledgling number	Causes of mortality
BC	BC:2	100	2	2	7	7	none
	BC:4	106	2	1	5	1	3 nestlings depredated; 1 unhatched egg
	BC:5	123	1	1	4	3	nestling mortality, cause unknown
SCR	SCR:1	130	2	0	7	0	both nests failed in early nestling stage
	SCR:5	106	1	1	2	2	none
UCI	UCI:3	75	1	0	4	0	adult bird depredated, nestlings die
	UCI:6	104	2	1	7	3	1st nest failed; 1 nestling depredated in 2nd
	UCI: 9	52	4	3	15	6	2nd nest failed in early nestling stage

### Nestling diet

Arthropod DNA was recovered from 28 of the 62 total fecal samples (45.1%), resulting in 326 OTUs at a 97% clustering threshold (S1 Table in S1 Appendix). The number of OTUs per sample ranged between 1 and 61 with a mean of 18.1 (±2.8 SE) and a median of 15 (S2 Fig in S1 Appendix). Using reference databases, we successfully assigned an arthropod order to 300 OTUs, resulting in 127 unique taxa with a mean percent similarity match of 97.3% (±0.13 SE) to the BOLD database (S1 Table in S1 Appendix). The majority of OTUs were identified to the genus-level or higher; 14.3% were resolved at the species-level, 55% to genus, 18.7% to family, and only the order was known for the remaining 12% (S1 Table in S1 Appendix). Fecal samples had a mean of 18.1 OTUs, 10.4 different taxa, and 3.6 arthropod orders.

**Fig 2:**
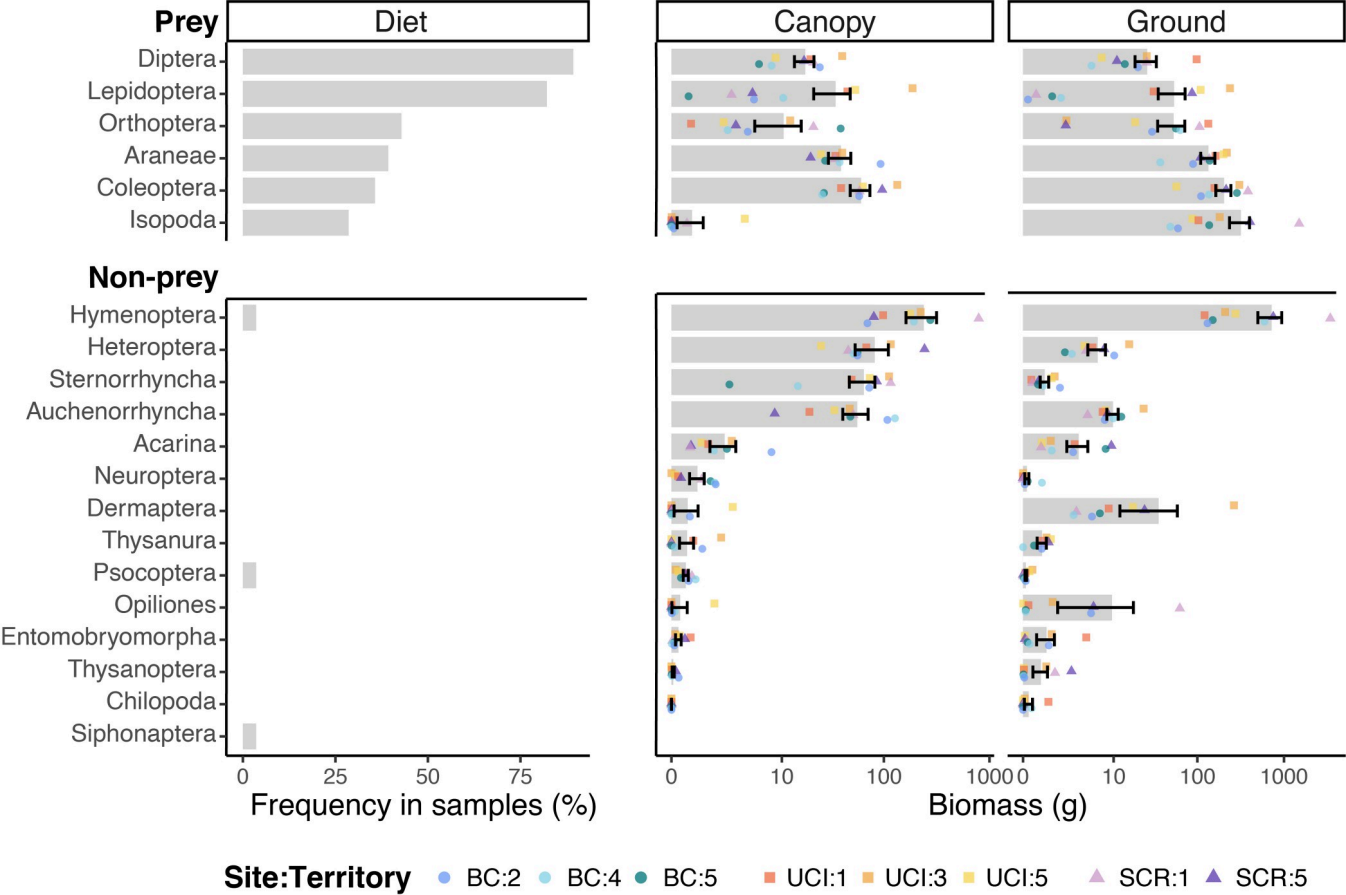
Arthropod orders and suborders in (a) Coastal Cactus Wren diet samples and (b) nesting territories. Arthropod taxa are ranked by the frequency of occurrence among diet samples. The total estimated biomass is given at the territory-scale for canopy and ground arthropods. Symbols show the variation in estimated biomass among territories (color) and site (shape). Grey bars show the mean biomass (+ SE) across all territories (n = 8). Biomass estimates are drawn on a log scale.

Diet analysis revealed 10 arthropod orders, 57 families, 76 genera and a minimum of 90 different prey species (S1 Table in S1 Appendix). Two arthropod orders–Diptera and Lepidoptera–represented most of the prey diversity and occurred in 89.3% and 82.1% of samples (Fig 2, S2 Fig in S1 Appendix), respectively, and the majority of OTUs across samples (S3 Fig in S1 Appendix). The taxa identified within these two orders were diverse, including 21 families of Diptera and 18 families of Lepidoptera (S1 Table in S1 Appendix). Within Diptera, Tipulidae (Crane flies), Syrphidae (Hoverflies), and Neridae (Cactus flies) were the most frequent among samples, whereas Erebidae (namely *Arachnis picta* and *Apantesis ursina*) were the most prevalent Lepidoptera taxa (S1 Table in S1 Appendix). Additionally, diet samples included, in order of decreasing frequency, Orthoptera, Araneae, Coleoptera, and Isopoda (Fig 2) which each occurred in between 28.5% and 42.9% of the diet samples. Relatively rare (less than 4% of diet samples; a single occurrence each) were Hymenoptera, Pscoptera, and Siphonaptera, and these orders were thus not classified as prey taxa in subsequent analyses.

### Field samples of arthropods

A total of 91,477 arthropods were collected from 1,360 ground and canopy samples, representing 22 orders and 3 suborders (of Hemiptera). Hymenoptera dominated arthropod biomass on the ground (46.9% of total biomass) while both Hymenoptera and Hemiptera (including Heteroptera, Sternorrhyncha, Auchenorrhyncha) were most numerous in plant canopies (40.1% and 34.7%, respectively) (Fig 1). At both strata, the Hymenoptera collected were dominated by a single species (>95%), the invasive ant, *Linepithema humile*. Hemipteran biomass was dominated by Auchenorrhyncha (leafhoppers, cicadas) and Heteroptera (true bugs) (Fig 2).

**Fig 3:**
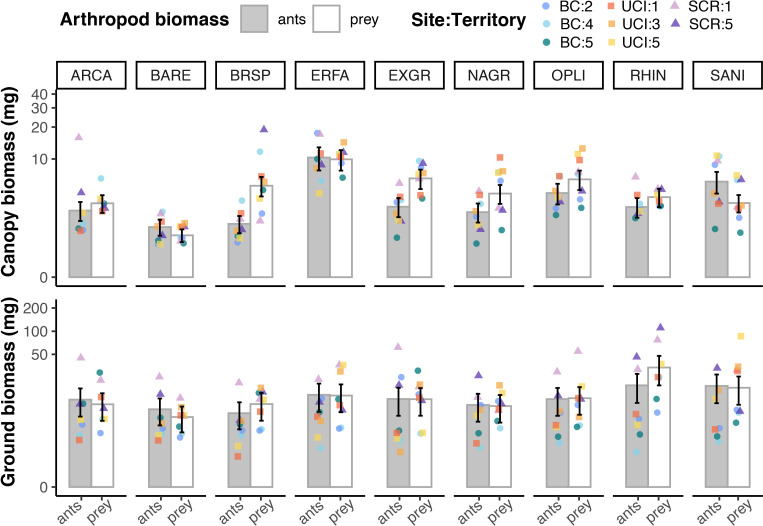
Territory- and habitat-element-specific estimates of arthropod density among habitat elements from canopy sampling (top row) and ground pitfall traps (bottom row). Grey bars show the biomass of non-native arthropods, Isopoda and Hymenoptera. White bars show the biomass of arthropod orders included in diet samples. Points are shown for each bird nesting territory, with shape referencing the study site and each territory a different color. Arthropods were sampled from native plant species; *Artemisia californica* (ARCA), *Erioginum*. *fasciculatum* (ERFA), *O*. *littoralis* (OPLI), *Rhus integrifolia* (RHIN), *Sambucus nigra* (SANI), and grasses (NAGR).; non-native *Brassica nigra* (BRSP) and grasses (EXGR); and bare ground (BARE). Bare ground was sampled by both pitfall sampling and vacuum collecting any arthropod observed within 0.5 m of the pitfall trap, referred to as canopy sampling above for consistency with plant sampling.

The total biomass of prey taxa (7 orders >98% of OTUs from diet; see above and Fig 2) and Hymenoptera biomass varied among habitat elements, territories, and sites as indicated by significant interactions between habitat element and territory (nested in site) for canopy and ground arthropods on both response variables (S2 Table in S1 Appendix). Across territories, *E*. *fasiculatum* canopies tended to be associated with the highest prey biomass, just above *Brassica nigra*, *O*. *littoralis*, and non-native grasses (Fig 3). In contrast, ground samples from beneath *Rhus integrifolia* tended to have more prey, however the biomass associated with this plant varied 10-fold among territories (Fig 3). Hymenoptera were most abundant in the canopies of *E*. *fasiculatum* and exceeded prey biomass on *Sambucus nigra*. Notably, Hymenoptera biomass was similar to that of prey biomass sampled across all habitat elements (in plant canopies and on the ground), with the exception of canopy sampled from *Brassica nigra* and grasses, which are estimated to support more prey biomass than ants (Fig 3).

**Fig 4:**
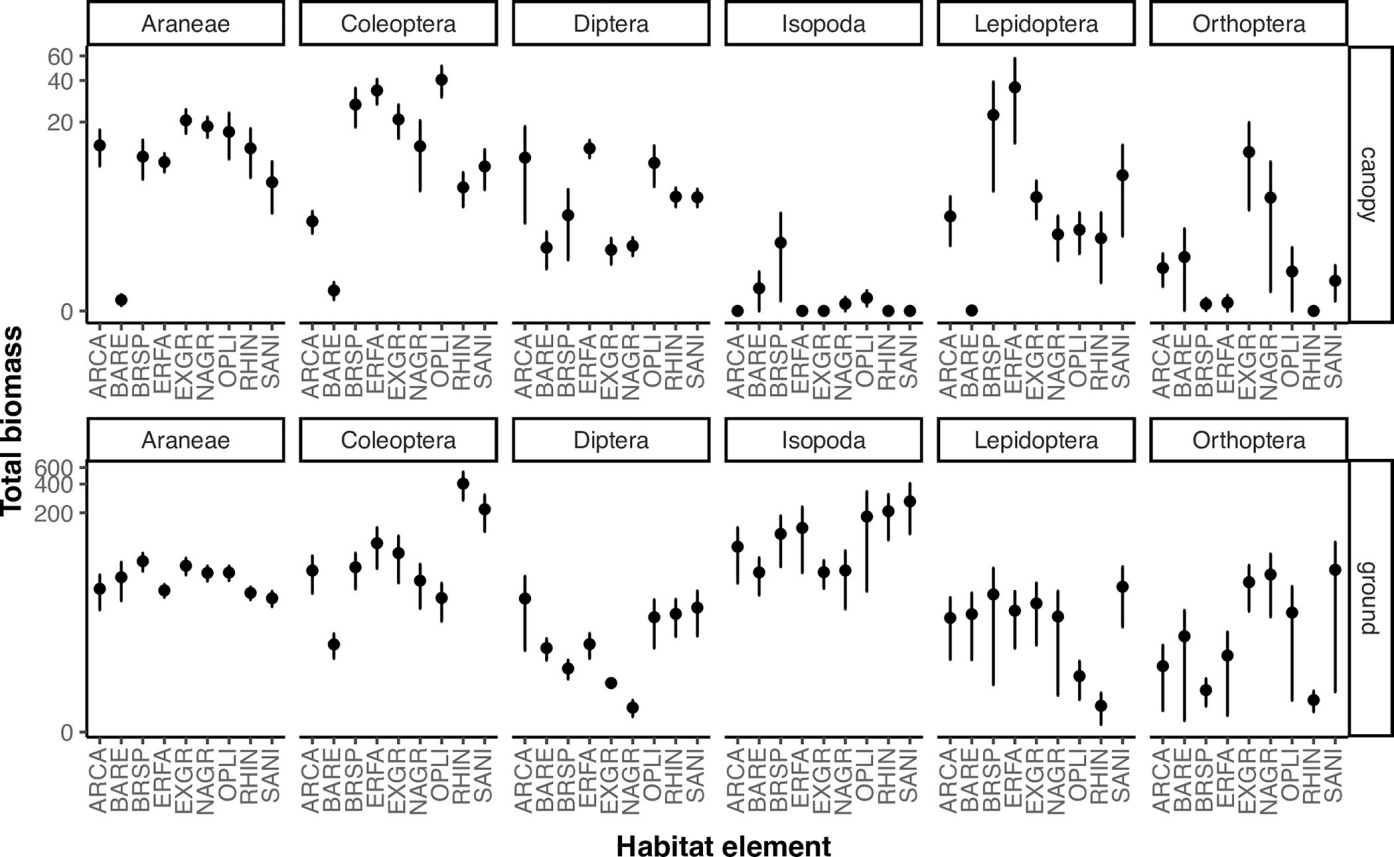
Mean biomass (mg ±SE) of prey orders from field-sampled arthropods across focal habitat elements in the canopy (top row) and on the ground (bottom row). Arthropods were sampled from native plant species; *Artemisia californica* (ARCA), *Eriogonum fasciculatum* (ERFA), *O*. *littoralis* (OPLI), *Rhus integrifolia* (RHIN), *Sambucus nigra* (SANI), and grasses (NAGR).; non-native *Brassica nigra* (BRSP) and grasses (EXGR); and bare ground (BARE). Bare ground was sampled by both pitfall sampling and vacuum collecting any arthropod observed within 0.5 m of the pitfall trap, referred to as canopy sampling above for consistency with plant sampling.

Moreover, arthropod composition differed among habitat elements in the canopy (PERMANOVA; P = 0.003) and on the ground (P = 0.001), as well as by site for both strata (canopy, P = 0.005; ground, P = 0.001) (Fig 4). In plant canopies Lepidoptera were most commonly sampled from *Brassica nigra* and *Eriogonum fasciculatum*; Coleoptera were associated with *Brassica nigra*, *Eriogonum fasciculatum*, grasses, and *O*. *littoralis;* Diptera were abundant on *Artemisia californica*, *Eriogonum fasciculatum*, and *O*. *littoralis;* Araneae were distributed evenly among habitat elements; grasses were the main source of Orthoptera (Fig 4). Arthropod prey sampled from the ground were dominated by Coleoptera and Isopoda (all non-native), in which *Rhus integrifolia and Sambucus nigra* were the primary sources for both orders, although Isopoda were common across all shrub species (Fig 4).

**Fig 5:**
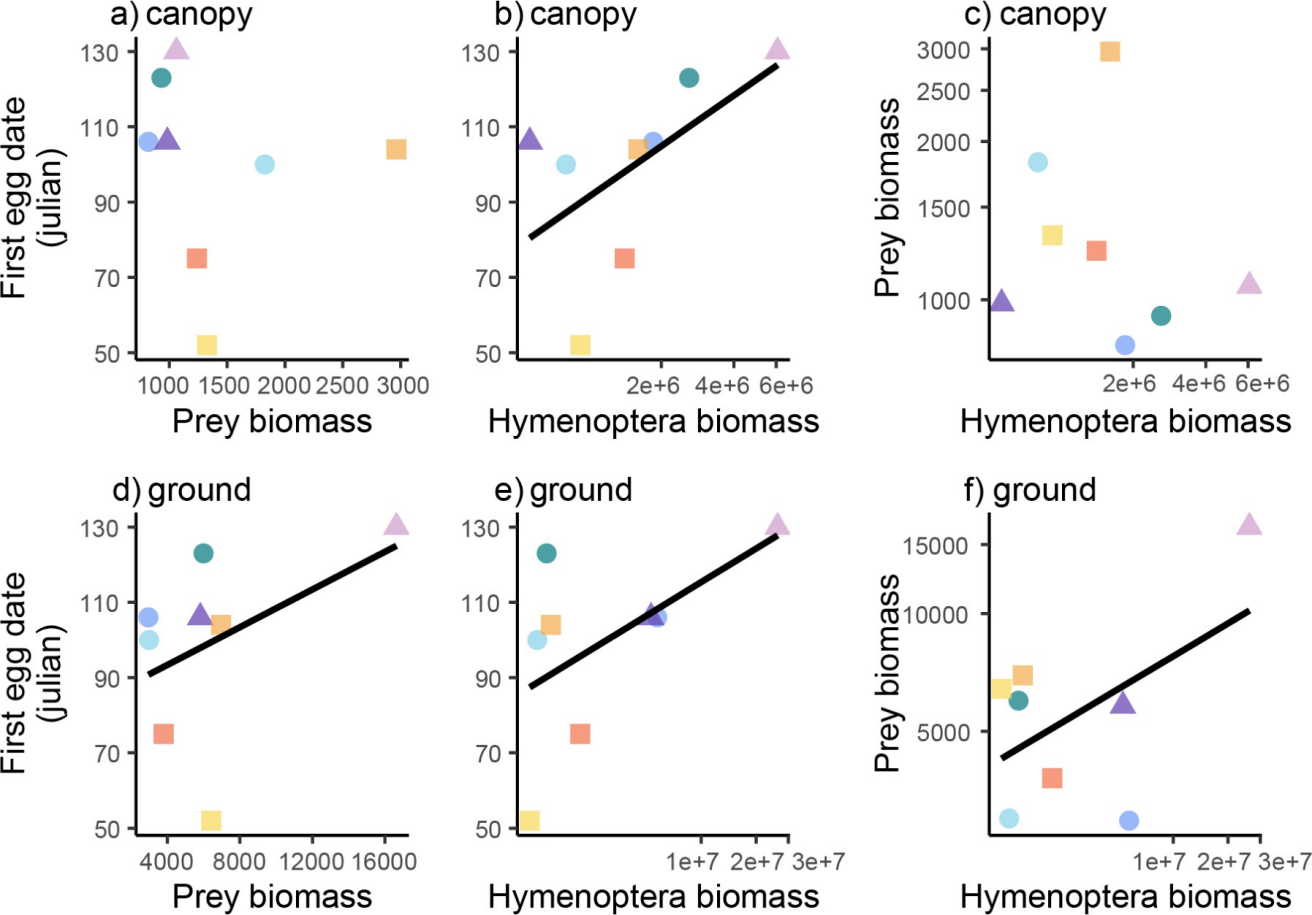
Relationships between the first egg date and territory-level estimates of prey (mg) (left column), first egg date and Hymenoptera biomass (mg) (middle column) and territory-level estimates of prey (mg) and Hymenoptera biomass (mg) (right column). Results presented for plant canopies (top row) and on the ground (bottom row).

Among territories, 49.8% of variation in canopy prey composition (PC1 canopy) was characterized by a trade-off between Lepidoptera biomass and the biomass of Araneae and Orthoptera (PC1 canopy, S4 Fig in S1 Appendix), with limited variation in the biomass of Diptera and Isopoda. Bivariate correlations indicated that in plant canopies, composition (PC1 canopy) was unrelated to both prey biomass (P = 0.14, R^2^ = 0.32) and Hymenoptera biomass (P = 0.55, R^2^ = 0.06). Similarly, there was no relationship between Hymenoptera and prey biomass in plant canopies (P = 0.72, R^2^ = 0.02) (Fig 5C). From ground sampling, 57% of variation in prey composition (PC1 ground) was driven by Isopoda, in which the relative biomass of Isopoda was negatively associated with all other orders (PC1 ground; S4 Fig in S1 Appendix). Subsequently, ground composition (PC1 ground) showed a positive relationship to overall prey biomass (P = 0.027, R^2^ = 0.54) and a positive relationship with Hymenoptera biomass on the ground (P = 0.007, R^2^ = 0.72), underlain by a marginally significant, positive, association between Hymenoptera and prey biomass from ground sampling (P = 0.055, R^2^ = 0.48) (Fig 5F).

### Plant communities

Approximately 83% of ground cover in nesting territories was vegetated, with half of the total area covered by native plant species (50.3% ± 6.21 SE) and 33.2% (± 5.13 SE) by non-native plant species (S5a Fig in S1 Appendix). The remaining area within territories was either bare (6.8% ± 0.94 SE) or contained artificial elements (8.6% ±3.9 SE) (e.g., golf range, landscaping, agriculture, or road). The focal plant species used in arthropod sampling were the majority of native plant cover, accounting for 40.6% of ground cover overall (± 4.91 SE), and *Brassica nigra* and non-native grasses were the primary non-native plants documented. Non-native grass cover was the most common vegetation across plots (23.9% ± 4.24 SE) followed by *Artemisia californica*, *Eriogonum fasciculatum*, and *Brassica nigra* (S5b Fig in S1 Appendix). Plant communities varied considerably among territories (S5a Fig in S1 Appendix); native plant cover ranged nearly 3-fold from 26% to 72% and non-native cover was between 18% and 56%. In particular, between 0 and 31% of ground cover was *Artemisia californica*, which was negatively related to non-native plant cover (P = 0.0247, R2 = 0.53).

### Relating arthropod communities, and nestling diet and timing of breeding

**Fig 6:**
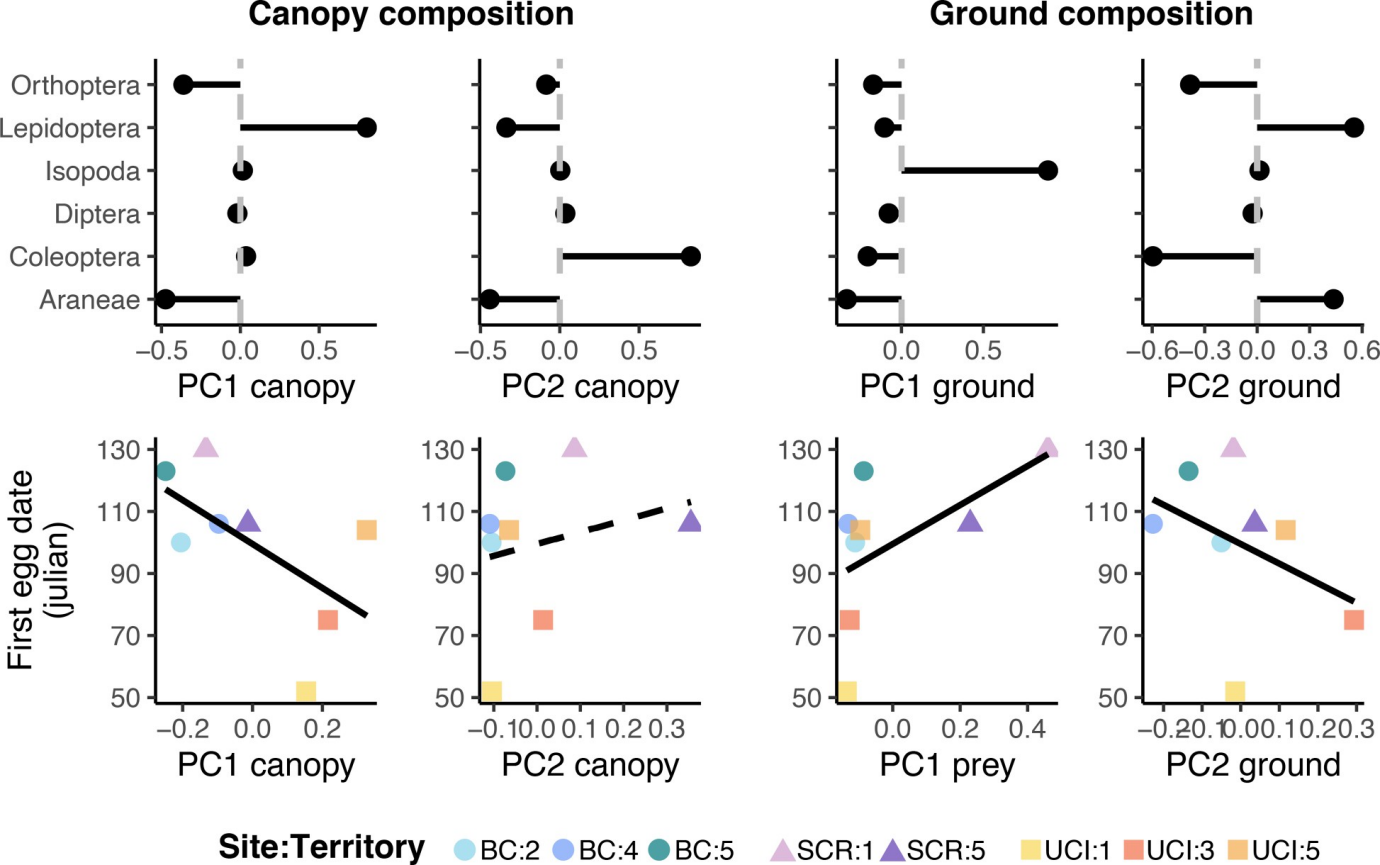
Territory-level relationships between arthropod prey composition and first egg date in plant canopies and on the ground. PC loadings are provided in the first row for PC axes representing variation in arthropod prey composition. Variation in composition is considered separately for canopy (columns 1 & 2) and ground (columns 3 & 4) arthropods. Trend lines indicate significant (P < = 0.05) correlation between axes. PC1 and PC2 for canopy composition explain 50% and 29% of variation and for ground composition explain 58% and 29% of variation (see S4 Fig in S1 Appendix).

Our tests relating territory-level estimates of arthropod biomass and composition to bird performance are based on correlations of small sample size (n = 8) and must thus be interpreted cautiously. First egg date was positively related to prey biomass on the ground (P = 0.003) such that territories with more ground-dwelling prey had delayed reproduction (Fig 4D). Conversely, there was no relationship between first egg date and canopy prey biomass (P = 0.48) (Fig 4A). Further, Hymenoptera biomass was positively related with the first egg date in plant canopies (P < 0.001) (Fig 5B) and on the ground (P < 0.001) (Fig 4E) indicating that birds reproduced later in the season when Hymenoptera were more abundant. These relationships are better understood via prey composition analyses. In plant canopies, prey composition (PC1 canopy) was negatively associated with the first egg date (P < 0.001) (Fig 6E) in which earlier reproduction aligned with more Lepidoptera biomass. On the ground, arthropod composition (PC1 ground) was positively associated with first egg date (P < 0.0001) (Fig 6G), in which Isopoda biomass corresponded to delayed reproduction.

## Discussion

This study links data on Coastal Cactus Wren nestling diets, arthropod availability in the environment and nesting performance to make inference on what aspects of arthropod community structure best support this threatened species. This task presents a significant challenge as inference relies on tests of territory-level co-variation between arthropods and Coastal Cactus Wren performance, and the challenges of characterizing arthropod communities at this scale constrain the territory sample size. Nevertheless, these analyses yield evidence for several compelling, testable hypotheses that are consistent with past studies of other systems. Specifically, we conclude that Coastal Cactus Wren nesting performance is limited by two effects of arthropod community structure, the availability of Lepidoptera and negative effects of the invasive arthropods *L*. *humile* and *A*. *vulgare*. We did not detect an effect of invasive arthropods on nestling food sources, suggesting they either had direct negative effects or affected food sources in ways we did not detect. Below we summarize our evidence for these hypotheses, propose the additional data required to test them, and consider the management implications.

We show a discordance between arthropod availability and nestling diet. While it is not clear if the observed diet reflects an optimal diet, it is apparent that most of what is in the environment is not consumed by nestlings. The observed diet consisted largely of Diptera and Lepidoptera (present in >75% of all samples), with lesser contributions of Orthoptera, Aranea, and Coleoptera and Isopoda (<50% of samples, each). With respect to the composition of the arthropod community, Diptera and Lepidoptera were substantially less abundant than other prey and non-prey taxonomic groups both from ground- and canopy-based sampling. In particular, Hemiptera (collectively, and each of the three sub-orders individually) were more abundant than any prey taxonomic group in canopy sampling. Similarly, Hymenoptera–consisting almost entirely of the invasive ant *L*. *humile*–was the most abundant arthropod taxa in both canopy and ground sampling but was not detected in nestling diets. Moreover, there is substantial within-order variation in prey quality that may mask associations with overall order biomass. These findings suggest that while arthropods may be abundant, the prey taxa for nestlings may nonetheless be rare, and an appropriate assessment of optimal food resources must take a nuanced approach.

We also find evidence that food limitation reduces Coastal Cactus Wren performance. The date of first egg laying is an accepted metric of performance, with early egg laying being positively correlated with other fitness metrics (S1 Fig in S1 Appendix; [51, 57, 58]). Among territories, egg laying day was earlier (i.e. stronger bird performance) in association with increases in the relative abundance (PC scores for community composition) of Lepidoptera (canopy Fig 5A–5C; ground Fig 5D–5F). This pattern is consistent with Lepidoptera being the most abundant taxa in nestling diet, and with many other studies documenting the importance of Lepidoptera in avian diets [71–73]. At the same time, egg laying date was later (i.e. poorer performance) in association with increases in the relative abundances of Coleoptera and Orthoptera for both canopy and ground sampling and with Araneae in canopy sampling (Fig 5). We speculate that these negative associations between performance and these three arthropod taxa is not due to any sort of direct negative effect, but rather to their trade-off with Lepidoptera, whose variation among territories was negatively associated with Coleoptera and Orthoptera for both canopy and ground sampling and with Araneae in canopy sampling only (S4 Fig in S1 Appendix). Inland populations of the cactus wren [56] demonstrated that supplementing nestling diet led to greater mass and survivorship of young birds, as well as increased parental reproductive output compared to food limited birds. Similarly, increased rainfall in arid systems is presumed to promote bird fecundity through bottom-up effects on plant productivity and food supply [74]. Food limitation has been demonstrated to affect wren productivity (number of young produced per year) in both coastal reserves [51] and in desert populations [54].

Contrary to expectations, total prey biomass at the territory level was not associated with an earlier egg laying date. We observed no association between prey biomass and egg laying date for canopy arthropods (Fig 5A). Surprisingly, there was a positive association between prey biomass and egg laying date (i.e. a negative effect on performance; Fig 6) for ground arthropods. We speculate that the significant relationship between Coastal Cactus Wren performance and prey community composition but not biomass was due to two related factors. First, some arthropod taxa may be consumed but are a suboptimal resource for developing birds, and variation in biomass of these groups may mask the influence of variation in biomass of taxa that drive performance. For example, the invasive species *A*. *vulgare* was in high relative abundance in ground arthropod communities, an abundant prey item, and negatively associated with bird performance (see below). Second, our assessment of arthropods (communities and nestling diet) at the ordinal level may mask important variation in the abundance of suitable prey if some but not all families are fed upon.

Our results also suggest that invasive arthropods reduce Coastal Cactus Wren performance, but the precise mechanism is unclear. Isopoda–which represented a significant component of the nestling diet (Fig 2)–consist nearly exclusively of the invasive species *A*. *vulgare*, and their relative abundance was associated with increased egg laying date (i.e. decreased performance; Fig 6). We speculate this effect was due either to toxicity as prey or perhaps to a general lack of nutritional value. Consistent with this view, Isopoda have been observed to carry and transmit avian parasites [48]. The mechanism by which *L*. *humile* affected egg laying date is less clear. Although *L*. *humile* is generally linked to loss of native arthropod populations in Coastal Sage Scrub habitat [75–77], within the territories we studied–all of which were invaded–we did not detect an association between variance in *L*. *humile* and prey abundance or composition. While L. *humile* can attack and harass nestlings [46] and has otherwise been observed to affect bird performance [78], the effect on date of first egg laying–our performance metric–cannot be explained by such dynamics unless adults perceived higher *L*. *humile* densities as being indicative of poor habitat quality and delayed egg laying. There was a strong positive association between *L*. *humile* and *A*. *vulgare* on the ground, and it is thus possible that some component of the negative effect of *L*. *humile* was mediated by their facilitating *A*. *vulgare*. *L*. *humile* invaded the region in the early 2000’s [79], displaced native ants [75] other native arthropods [76, 77] and may thus have played a role in *A*. *vulgare* invasion.

Our hypotheses for the influence of arthropod community structure on Coastal Cactus Wren performance in turn begs the question of what territory attributes are likely to yield the most beneficial arthropod communities. With respect to the invasive arthropods, variation in abundance was not associated with plant community composition in either ground or canopy sampling (*L*. *humile* see Fig 3; *A*. *vulgare* see Fig 4). *L*. *humile* abundance varied among plant taxa in proportion to variation in other arthropods for both canopy and ground sampling. At the same time, *A*. *vulgare* was rare in canopies and present at a relatively consistent abundance in ground sampling. Accordingly, it appears that vegetation composition does not exert a meaningful influence on these invasive arthropods and, as a result, alteration of plant community composition does not offer an effective management strategy. In contrast, the abundance of *L*. *humile* is known to be associated with irrigation and other habitat modifications that provide more humid nesting sites [80]. Similarly, terrestrial isopods are generally believed to be limited by moisture availability, due in part to their recent evolutionary history of occupying aquatic and littoral habitats [81]. Increased moisture is typically associated with irrigated habitat edges, emphasizing the importance of minimizing habitat fragmentation.

With respect to caterpillar (lepidopteran larvae) abundance, we observed substantial variation among plants taxa. Density was higher in the canopies of *Eriogonum fasciculatum*, *Sambucus nigra*, and *Brassica nigra* and was particularly rare on the ground beneath *Opuntia littoralis* and *Rhus integrifolia*. With respect to *Brassica nigra*, the only caterpillars occurring are of *Pieris* species (Pieridae), and these were not observed in the Coastal Cactus Wren diet (S1 Table in S1 Appendix). Accordingly, we predict that Coastal Cactus Wren performance should be highest in territories with abundant caterpillar-yielding plant taxa, i.e. *Eriogonum fasciculatum*, *Sambucus nigra (*but not *Brassica nigra)*. If additional evidence supported this prediction, management activities could respond accordingly.

In summary, our results suggest several testable hypotheses for the habitat attributes likely to promote Coastal Cactus Wren performance. Coastal Cactus Wren performance was improved by the availability of key prey items (Lepidopteran and Diptera), which were most strongly associated with *Eriogonum fasciculatum* and *Sambucus nigra*. Performance was in turn reduced by two invasive arthropods associated with moisture sources. There are several means by which evidence for these hypotheses might be more rigorously tested. First, the sample sizes (territory number) for associating bird performance with arthropods could be increased by focusing sampling on prey (Lepidoptera) and invasive arthropods (*L*. *humile* and *A*. *vulgare*). Tailoring sampling methods for just these taxa would simultaneously increase resolution and efficiency. In addition, territories could be intentionally selected to include variation in invasive taxa based upon habitat aridity or other factors. In this regard, repeated measures time series data might be especially valuable, as tests for the effects of year-to-year variation could test for the influence of arthropod communities on Coastal Cactus Wren performance while controlling for other potentially influential factors that vary among territories (e.g. proximity to human disturbance and nest predators). Further evidence for the impact of *L*. *humile* could also come from the experimental ant removal at the territory level and for the impact of *A*. *vulgare* through tests for the associated parasites.

## Supporting information

S1 Appendix This file contains all the supporting tables and figures. (PDF)
