# Supplementary material for: Consequences of arthropod community structure for an at-risk insectivorous bird
Source: PLoS One. 2023 Feb 10;18(2):e0281081. doi: 10.1371/journal.pone.0281081 (PMC9917275; doi:10.1371/journal.pone.0281081)
Supplement: S1 Appendix — (PDF) [file pone.0281081.s001.pdf]

Appendix

Table S1. Arthropods identified in diet from DNA barcoding of fecal samples. The number of OTUs associated with each classification, the mean percent similarity match to the BOLD database, and the frequency of occurrence from the fecal samples are given. Rows shaded in grey are the aggregate for all OTUs at the order-level while rows below detail the resolved taxonomy within the respective order.

**Taxonomy**					**# OTUs**	**BOLD similarity**	**Frequency in Samples (%)**
Class	Order	Family	Genus	Species			
**Arachnida**	**Araneae**				**47**	**96.85**	**39.29**
Arachnida	Araneae	Araneidae	Argiope	trifasciata	1	99.36	3.57
Arachnida	Araneae	Araneidae	Cyclosa	turbinata	2	99.61	25
Arachnida	Araneae	Araneidae	Cyclosa		27	97.08	14.29
Arachnida	Araneae	Araneidae			11	93.82	10.71
Arachnida	Araneae	Salticidae	Habronattus	clypeatus	1	100	3.57
Arachnida	Araneae	Salticidae	Habronattus		1	96.75	3.57
Arachnida	Araneae	Salticidae	Phidippus	boei	1	99.36	3.57
Arachnida	Araneae	Salticidae	Phidippus	johnsoni	1	99.36	10.71
Arachnida	Araneae				2		7.14
**Insecta**	**Blattodea**				**7**	**97.46**	**25**
Insecta	Blattodea	Ectobiidae			6	97.71	21.43
Insecta	Blattodea				1	95.4	7.14
**Insecta**	**Coleoptera**				**20**	**98.07**	**39.29**
Insecta	Coleoptera	Anthicidae			1	99.19	7.14
Insecta	Coleoptera	Carabidae	Acupalpus	testaceus	1	99.4	7.14
Insecta	Coleoptera	Carabidae	Calathus	ruficollis	1	100	10.71
Insecta	Coleoptera	Carabidae			2	95.26	10.71
Insecta	Coleoptera	Chrysomelidae	Glyptina	atriventris	1	100	7.14
Insecta	Coleoptera	Chrysomelidae	Glyptina		1	96.75	7.14
Insecta	Coleoptera	Curculionidae	Anthonomus		2	98.22	10.71
Insecta	Coleoptera	Curculionidae	Celebia		1	98.77	7.14
Insecta	Coleoptera	Elateridae	Melanotus		1	98.08	7.14
Insecta	Coleoptera	Lampyridae	Pyropyga	nigricans	2	97.58	7.14
Insecta	Coleoptera	Melyridae			1	98.7	7.14
Insecta	Coleoptera	Ptinidae			1	100	7.14
Insecta	Coleoptera	Scarabaeidae			2	97.66	10.71
Insecta	Coleoptera	Tenebrionidae	Tenebrio	molitor	1	100	17.86
Insecta	Coleoptera				2	94.66	7.14
**Insect**	**Diptera**				**86**	**97.30**	**89.29**
Insecta	Diptera	Acrididae			1	93.49	14.29
Insecta	Diptera	Anthomyiidae	Hydrophoria		1	97.65	10.71
Insecta	Diptera	Bombyliidae	Bombylius		1	97.18	7.14
Insecta	Diptera	Bombyliidae			4	97.55	10.71
Insecta	Diptera	Cecidomyiidae			3	97.37	10.71
Insecta	Diptera	Ceratopogonidae	Culicoides		1	98.29	7.14

Insecta	Diptera	Ceratopogonidae			1	98.08	7.14
Insecta	Diptera	Chironomidae	Krenopelopia		1	96.05	7.14
Insecta	Diptera	Chloropidae	Dasyopa	triangulata	1	98.89	7.14
Insecta	Diptera	Chloropidae	Thaumatomyi a	annulata	1	100	7.14
Insecta	Diptera	Chloropidae	Thaumatomyia		1	95.51	7.14
Insecta	Diptera	Dolichopodidae	Condylostylus		2	97.62	14.29
Insecta	Diptera	Drosophilidae	Drosophila		1	95.06	7.14
Insecta	Diptera	Ephydridae			1	100	14.29
Insecta	Diptera	Heleomyzidae			1	98.72	7.14
Insecta	Diptera	Limoniidae			1	97.44	7.14
Insecta	Diptera	Neriidae	Odontoloxozus	pachycericola	1	100	21.43
Insecta	Diptera	Neriidae	Odontoloxozus		4	96.5	7.14
Insecta	Diptera	Neriidae			1	93.96	7.14
Insecta	Diptera	Phoridae	Megaselia		1	97.21	7.14
Insecta	Diptera	Scathophagidae	Scathophaga		1	95.79	7.14
Insecta	Diptera	Sciaridae	Claustropyga		1	95.06	7.14
Insecta	Diptera	Sciaridae			3	95.52	50
Insecta	Diptera	Stratiomyidae	Sargus		2	95.06	10.71
Insecta	Diptera	Syrphidae	Cheilosia		1	96.3	7.14
Insecta	Diptera	Syrphidae	Copestylum	avidum	1	99.36	14.29
Insecta	Diptera	Syrphidae	Copestylum	mexicanum	1	99.36	17.86
Insecta	Diptera	Syrphidae	Copestylum		3	97.81	10.71
Insecta	Diptera	Tabanidae	Hybomitra	pechumani	1	100	7.14
Insecta	Diptera	Tabanidae	Hybomitra		1	98.72	7.14
Insecta	Diptera	Tachinidae	Anisia		1	96.05	7.14
Insecta	Diptera	Tachinidae	Archytas	apicifer	1	100	7.14
Insecta	Diptera	Tachinidae	Ceracia		3	98.05	10.71
Insecta	Diptera	Tachinidae	Exoristoides		1	98.72	7.14
Insecta	Diptera	Tachinidae	Myiopharus		1	97.5	7.14
Insecta	Diptera	Tachinidae	Periscepsia		5	98.35	25
Insecta	Diptera	Tachinidae			1	97.75	10.71
Insecta	Diptera	Tipulidae	Holorusia		1	100	17.86
Insecta	Diptera	Tipulidae	Tipula	oleracea	1	99.35	7.14
Insecta	Diptera	Tipulidae	Tipula		8	98.5	14.29
Insecta	Diptera	Tipulidae			1	98.08	7.14
Insecta	Diptera				15	95.51	42.86
**Insecta**	**Hymenopter a**				**1**	**92.31**	**7.14**
Insecta	Hymenoptera	Braconidae			1	92.31	7.14
**Insecta**	**Lepidoptera**				**104**	**98.55**	**82.14**
Insecta	Lepidoptera	Acrolophidae	Acrolophus	kearfotti	2	99.35	7.14
Insecta	Lepidoptera	Acrolophidae	Acrolophus		2	97.77	7.14
Insecta	Lepidoptera	Apateloididae	Apatelodes		1	97.3	7.14
Insecta	Lepidoptera	Autostichidae	Oegoconia		2	97.62	7.14
Insecta	Lepidoptera	Coleophoridae	Coleophora		1	95.45	7.14
Insecta	Lepidoptera	Erebidae	Apantesis	ursina	5	99.82	35.71
Insecta	Lepidoptera	Erebidae	Apantesis		4	98.02	25
Insecta	Lepidoptera	Erebidae	Arachnis	picta	23	98.27	50
Insecta	Lepidoptera	Erebidae	Orgyia	vetusta	1	99.36	17.86
Insecta	Lepidoptera	Erebidae	Orgyia		2	97.48	7.14

Insecta	Lepidoptera	Erebidae	Rhapsa		1	98.9	7.14
Insecta	Lepidoptera	Erebidae	Spilosoma		1	97.83	7.14
Insecta	Lepidoptera	Erebidae	Trichromia		1	99.11	7.14
Insecta	Lepidoptera	Erebidae			2	96.92	14.29
Insecta	Lepidoptera	Gelechiidae	Monochroa	placidella	2	97.8	7.14
Insecta	Lepidoptera	Gelechiidae			1	97.53	7.14
Insecta	Lepidoptera	Geometridae	Glaucina		1	99.36	7.14
Insecta	Lepidoptera	Geometridae	Idaea		1	96.39	7.14
Insecta	Lepidoptera	Geometridae	Pero	mizon	1	100	10.71
Insecta	Lepidoptera	Geometridae	Prochoerodes		1	98.72	7.14
Insecta	Lepidoptera	Lycaenidae	Euphilotes	mojave	1	99.35	7.14
Insecta	Lepidoptera	Lycaenidae	Euphilotes		3	98.38	10.71
Insecta	Lepidoptera	Noctuidae	Acontia		1	96	7.14
Insecta	Lepidoptera	Noctuidae	Hemieuxoa	rudens	2	99.35	7.14
Insecta	Lepidoptera	Noctuidae	Lacinipolia		2	99	10.71
Insecta	Lepidoptera	Noctuidae	Peridroma	saucia	2	99.32	7.14
Insecta	Lepidoptera	Noctuidae	Platypolia		1	98.68	7.14
Insecta	Lepidoptera	Noctuidae	Protoschinia	scutosa	2	98.05	7.14
Insecta	Lepidoptera	Noctuidae	Schinia		1	98.92	7.14
Insecta	Lepidoptera	Noctuidae	Spodoptera		1	98.54	7.14
Insecta	Lepidoptera	Noctuidae	Trichoplusia		3	98.5	7.14
Insecta	Lepidoptera	Notodontidae	Elymiotis	notodontoides	1	97.7	7.14
Insecta	Lepidoptera	Nymphalidae	Hyposcada	illinissa	1	100	10.71
Insecta	Lepidoptera	Nymphalidae	Napeogenes		1	98.72	14.29
Insecta	Lepidoptera	Oecophoridae			1	100	7.14
Insecta	Lepidoptera	Papilionidae	Papilio		1	98.9	14.29
Insecta	Lepidoptera	Pterophoridae			1	93.83	7.14
Insecta	Lepidoptera	Pyralidae	Ozamia	fuscomaculella	5	98.92	14.29
Insecta	Lepidoptera	Pyralidae	Toripalpus	trabalis	1	99.26	7.14
Insecta	Lepidoptera	Tortricidae	Acleris		1	98.88	7.14
Insecta	Lepidoptera	Tortricidae	Epagoge		1	98.85	7.14
Insecta	Lepidoptera	Ypsolophidae	Ypsolopha		1	95.4	7.14
Insecta	Lepidoptera	Ypsolophidae			1	95.4	7.14
Insecta	Lepidoptera				14	98.47	50
**Insecta**	**Orthoptera**				**26**	**96.20**	**42.86**
Insecta	Orthoptera	Acrididae	Chloealtis		1	95.48	17.86
Insecta	Orthoptera	Acrididae			5	93.49	14.29
Insecta	Orthoptera	Gryllidae	Gryllodes	supplicans	2	100	35.71
Insecta	Orthoptera	Gryllidae	Gryllodes		9	96.51	7.14
Insecta	Orthoptera	Gryllidae			1	96.51	7.14
Insecta	Orthoptera	Tettigoniidae	Aglothorax		1	96.15	14.29
Insecta	Orthoptera	Tettigoniidae			2	93.98	3.57
Insecta	Orthoptera				5	91.16	10.71
**Insecta**	**Psocodea**				**1**	**100**	**7.14**
Insecta	Psocodea	Dasydemellidae	Teliapsocus	conterminus	1	100	7.14
**Insecta**	**Siphonaptera**				**2**	**97.66**	**7.14**
Insecta	Siphonaptera	Pulicidae	Echidnophaga	gallinacea	2	97.66	7.14
**Malacostraca**	**Isopoda**				**6**	**98.54**	**32.14**

Malacostraca	Isopoda	Armadillidiidae	Armadillidium	vulgare	6	98.54	32.14

Table S2. Summary of linear mixed model fixed effects evaluating variation in prey and Hymenoptera biomass from canopy and ground arthropod sampling. Models include habitat element, site, territory nested within site, and all interactions as fixed effects. Experimental block and sampling period were included as random effects. Hymenoptera biomass was log-transformed for normality of residuals. Significant (P < 0.05) and marginally significant (0.05 < P < 0.10) effects are in bold and italics, respectively.

**Response**	**Term**	**Chisq**	**df**	**P**
Prey in canopy	(Intercept)	11.78	1	**0.0006**
	Habitat element	26.75	8	**0.0008**
	Site	0.04	2	0.9810
	Habitat element:Site	18.62	16	0.2888
	Habitat element:Site:Territory	66.79	43	**0.0115**
Prey on ground	(Intercept)	30.43	1	**0.0000**
	Habitat element	16.99	8	**0.0302**
	Site	5.49	2	*0.0642*
	Habitat element:Site	41.46	16	**0.0004**
	Habitat element:Site:Territory	67.47	43	**0.0099**
Hymenoptera in canopy	(Intercept)	2.32	1	**0.1281**
	Habitat element	24.76	8	**0.0017**
	Site	15.53	2	**0.0004**
	Habitat element:Site	22.18	16	0.1374
	Habitat element:Site:Territory	59.74	43	**0.0462**
Hymenoptera on ground	(Intercept)	23.92	1	**0.0000**
	Habitat element	9.95	8	0.2684
	Site	13.38	2	**0.0012**
	Habitat element:Site	22.87	16	**0.1172**
	Habitat element:Site:Territory	86.68	43	**0.0001**

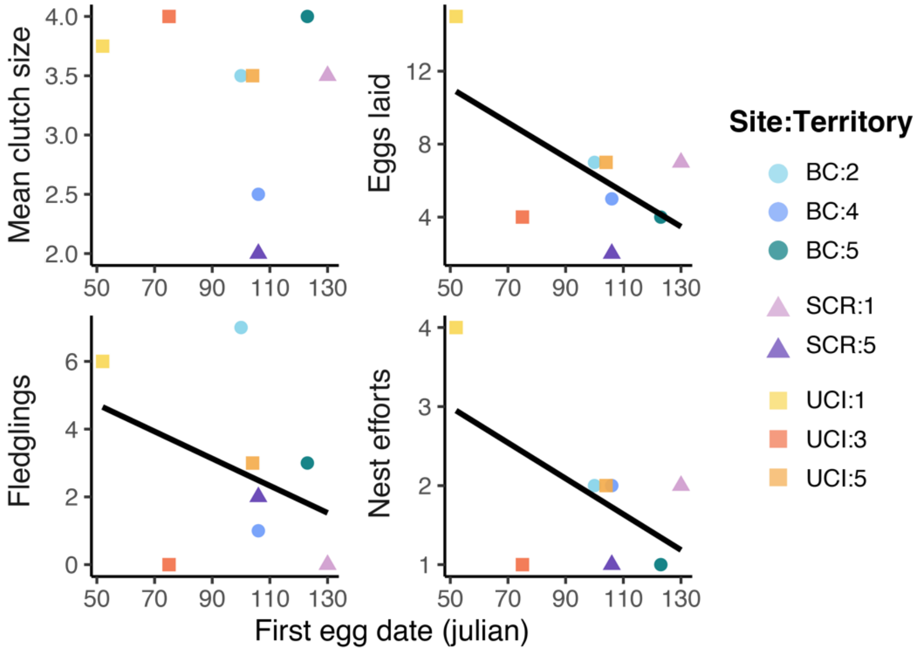

Figure S1. Relationships between the first egg date and mean clutch size (P = 0.13), total eggs laid (P < 0.001), number of fledglings (P = 0.004), and nest efforts (P < 0.001) of nesting territories in 2012.

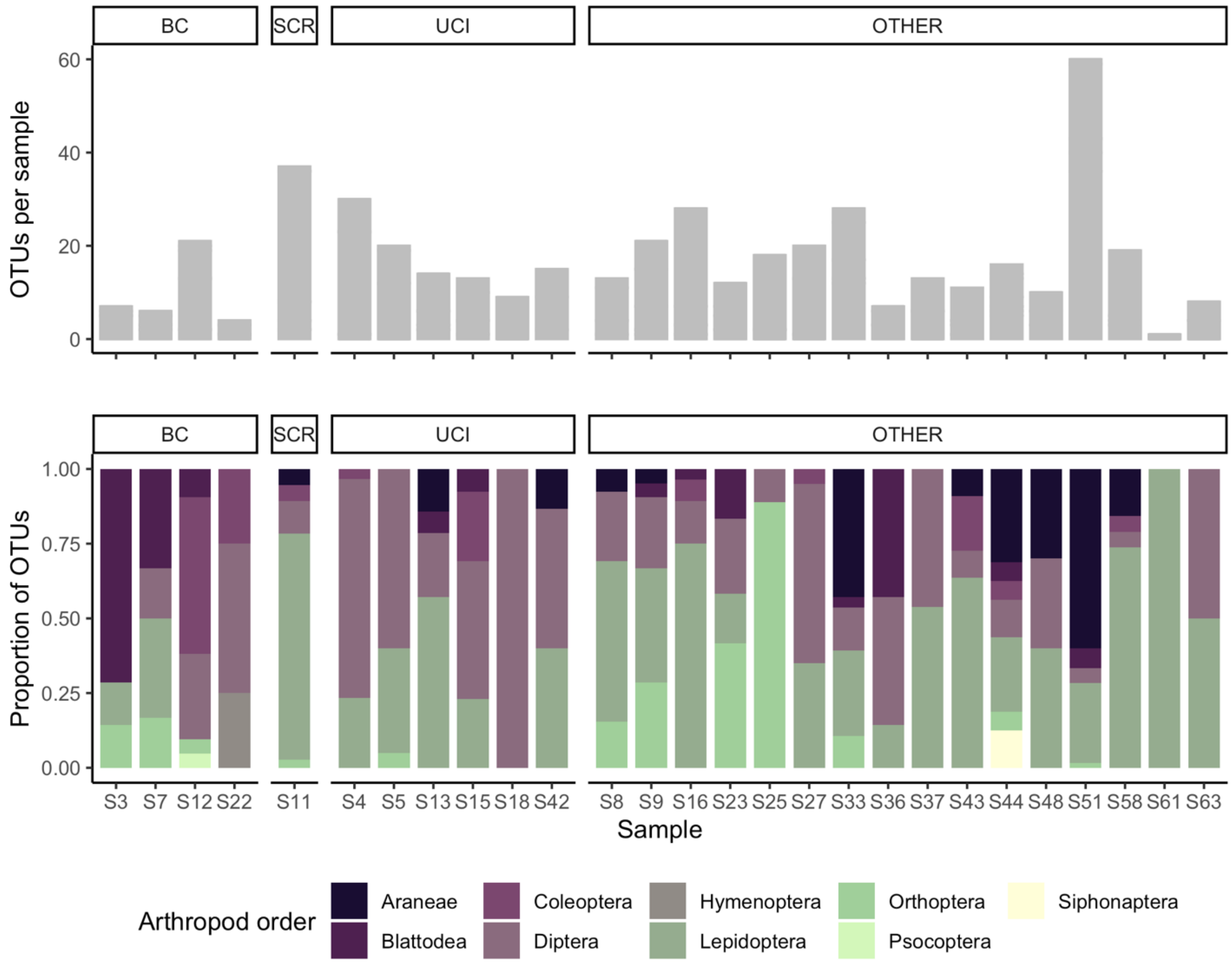

Figure S2. Total OTU richness (top) and taxonomic composition (bottom) from fledgling fecal samples. Taxonomic composition is the proportion of OTUs in each sample by arthropod order. Samples are grouped by the site collected from, 'OTHER' indicates samples that came from sites or time periods outside of the primary study.

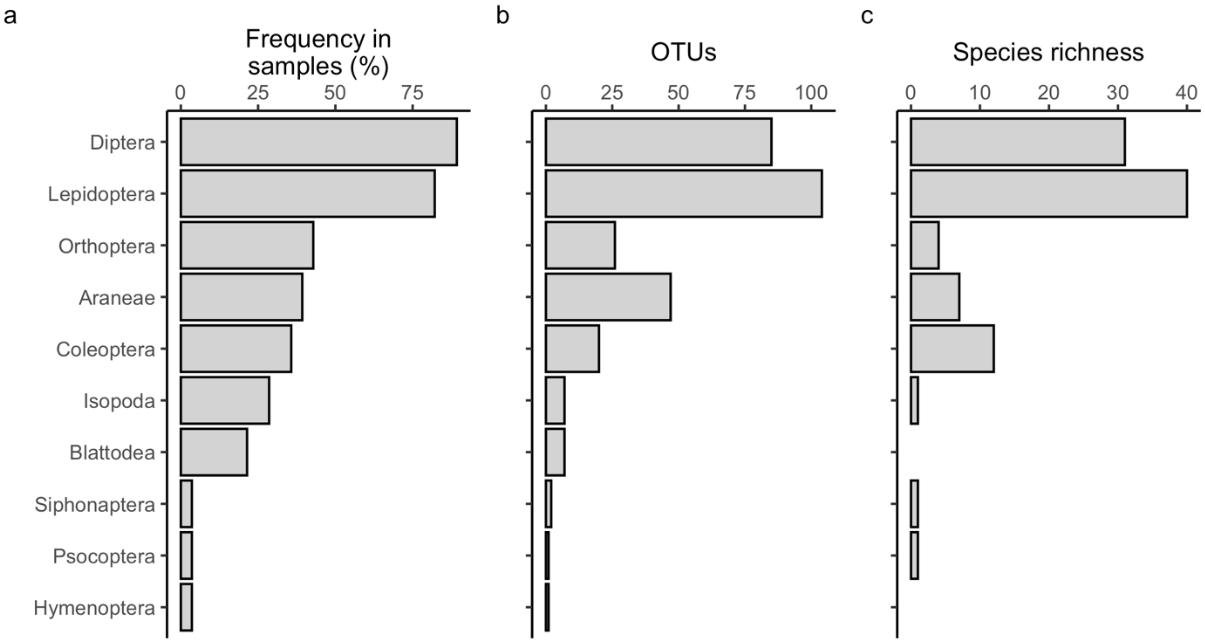

Figure S3. Diversity of arthropod prey from Coastal Cactus Wren diet analysis. The (a) frequency of occurrence in samples from Fig. 1 is shown alongside the (b) number of unique OTUs detected (b), and species richness (c) for each arthropod order. Species richness is the minimum number of possible species given variation in taxonomic rank among ids.

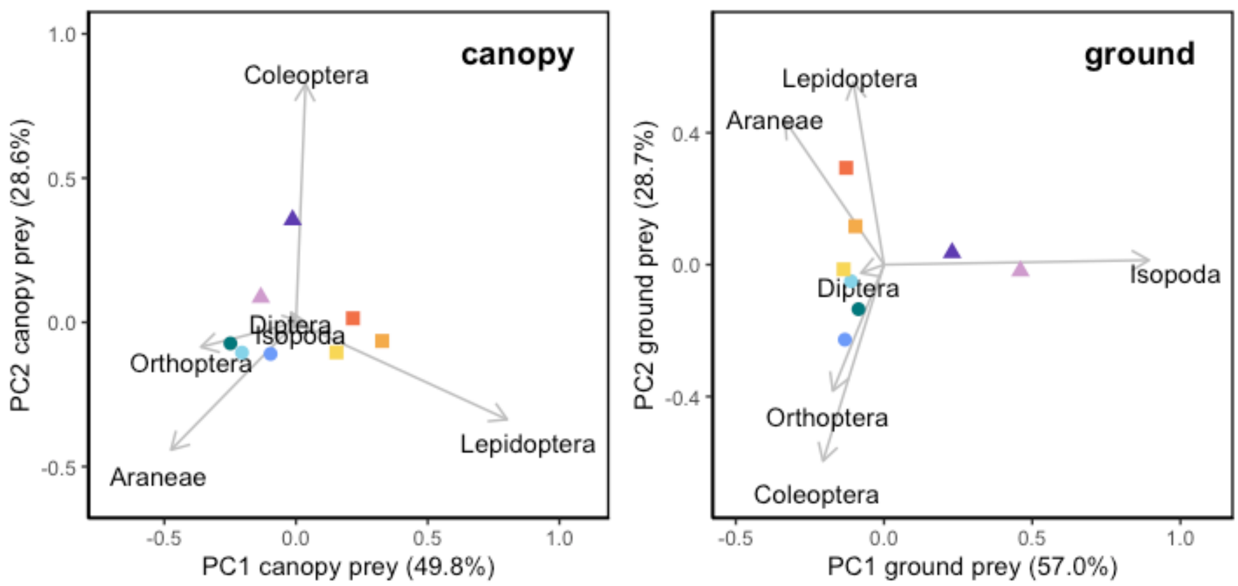

Figure S4. Variation in arthropod prey composition among nesting territories in plant canopies (left) and on the ground (right). All PCA ordinations are based on the relative biomass of prey orders identified in diet; Araneae, Coleoptera, Diptera, Isopoda, Lepidoptera, Orthoptera. Grey biplot arrows reflect the variable loadings for each order used in the PCA. Legend symbols are consistent with previous figures.

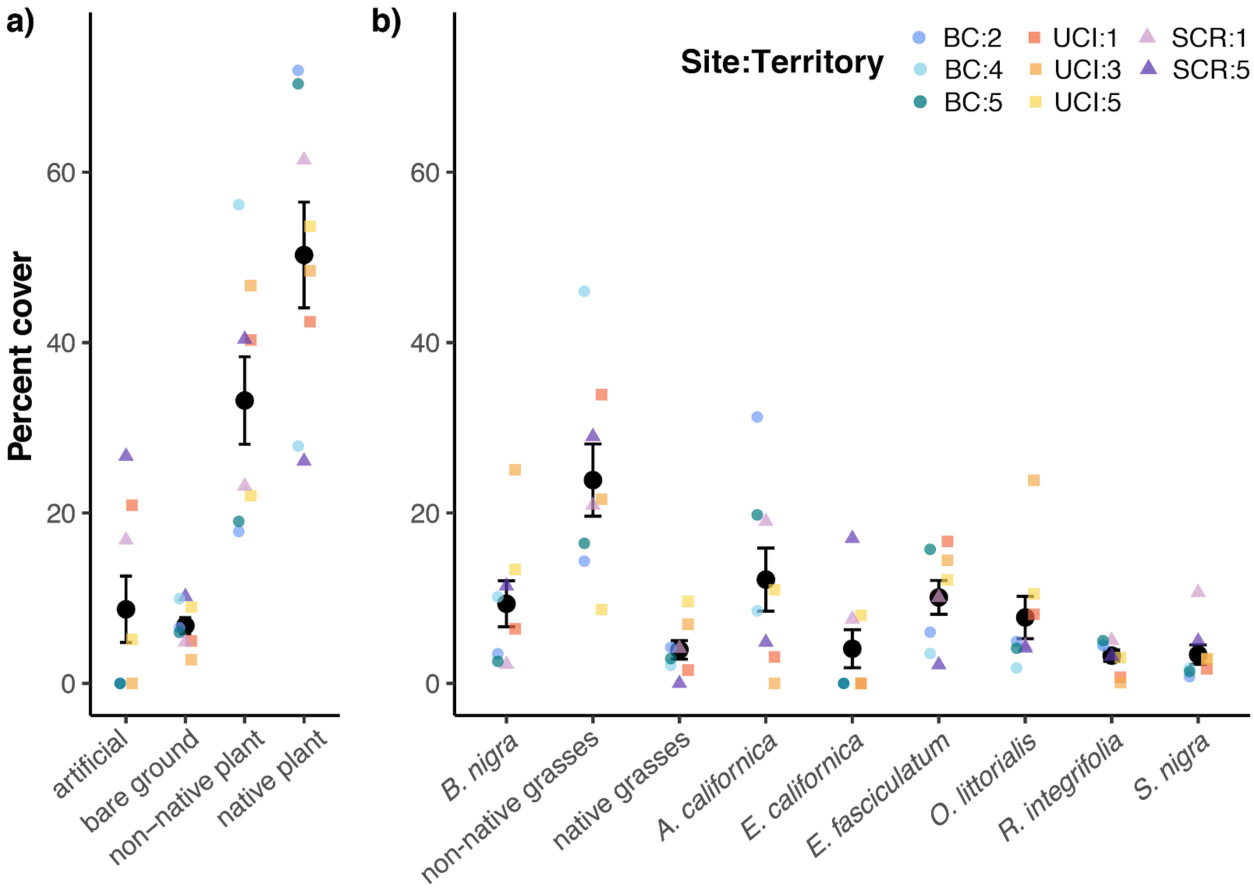

Figure S5. Nesting territory (a) ground cover and (b) vegetation cover of focal habitat elements. Mean percent cover across territories (+ SE) is shown in black; colored points reflect among-territory variation in cover. Artificial cover includes manipulated landscapes including golf course, agriculture, roads, and other development. *Brassica nigra* and non-native grasses reflect non-native plant cover. Native plants include focal habitat elements *Artemisia californica, E. californica, Eriogonum fasciculatum, Opuntia littorialis, Rhus integrifolia,* and *Sambucus nigra,* in addition to other, less common, native taxa documented.
